# Development and characterization of *Citrus limon* (L.) Osbeck essential oil nanoemulsions: assessment of antibacterial and antifungal activities

**DOI:** 10.22034/cmm.2025.345248.1633

**Published:** 2025-02-01

**Authors:** Iman Haghani, Mahdi Abastabar, Mahmoud Osanloo, Ali Davoodi, Amirhossein Ghasemzadeh, Masoumeh Eslamifar, Emran Habibi, Javad Akhtari

**Affiliations:** 1 Invasive Fungi Research Center, Communicable Diseases Institute, Mazandaran University of Medical Sciences, Sari, Iran; 2 Department of Medical Mycology, School of Medicine, Mazandaran University of Medical Sciences, Sari, Iran; 3 Department of Medical Nanotechnology, School of Advanced Technologies in Medicine, Fasa University of Medical Sciences, Fasa, Iran; 4 Medicinal Plants Research Center, Mazandaran University of Medical Sciences, Sari, Iran; 5 Student Research Committee, School of Medicine, Mazandaran University of Medical Sciences, Sari, Iran; 6 Department of Environmental Health Engineering, Faculty of Health, Mazandaran University of Medical Sciences, Sari, Iran; 7 Centre for Natural Products Discovery, School of Pharmacy and Biomolecular Sciences, Liverpool John Moores University, Liverpool L3 3AF, UK; 8 Molecular and Cell Biology Research Center, School of Medicine, Mazandaran University of Medical Sciences, Sari, Iran; 9 Department of Medical Nanotechnology, School of Advanced Technologies in Medicine, Mazandaran University of Medical Sciences, Sari, Iran

**Keywords:** *Citrus limon* (L.) Osbeck, Essential oil, Liposome, Nano, Nanoemulsion

## Abstract

**Background and Purpose:**

Essential oils (EO) have gained significant attention due to their natural antimicrobial and antifungal properties. However, their application is often limited due to poor solubility, volatility, and stability. Nanoemulsions, as advanced delivery systems, can overcome these limitations by enhancing the bioavailability and efficacy of EOs. Lemon EO, known for its broad-spectrum antimicrobial activity, is a promising candidate for nanoemulsion formulation. This study aimed to synthesize and characterize lemon EO nanoemulsions and evaluate their enhanced antimicrobial and antifungal potential, compared to crude oil.

**Materials and Methods:**

Lemon EO was first analyzed using gas chromatography-mass spectrometry (GC-MS) to identify its chemical composition. Lemon EO nanoemulsions were prepared using the spontaneous emulsification technique. The physicochemical properties, including particle size, polydispersity index (PDI), zeta potential, and stability, were characterized using dynamic light scattering. The antimicrobial and antifungal activities were assessed against *Escherichia coli*, *Staphylococcus aureus*, *Staphylococcus epidermidis*, *Enterococcus faecalis*, *Candida albicans*, and *Aspergillus fumigatus* through minimum inhibitory concentration (MIC) and minimum bactericidal/fungicidal concentration (MBC/MFC) assays.

**Results:**

The GC-MS analysis revealed the major chemical components of lemon EO, including limonene, β-pinene, and γ-terpinene. The nanoemulsions exhibited a mean particle size of about 15 nm, a low PDI (< 0.3), and a negative zeta potential, indicating high stability and homogeneity. The antimicrobial and antifungal activities of the nanoemulsions were significantly enhanced compared to the crude lemon EO, as demonstrated by lower MIC and MFC values. The nanoemulsions also showed excellent stability under various storage conditions.

**Conclusion:**

This study demonstrated that lemon EO nanoemulsions are a stable delivery system with superior antimicrobial and antifungal properties. The GC-MS analysis provided valuable insights into the chemical composition of the EO, further supporting its efficacy. These findings suggest the potential of lemon EO nanoemulsions as a natural alternative for applications in food preservation, pharmaceuticals, and cosmetics.

## Introduction

The increasing prevalence of antimicrobial resistance has necessitated the exploration of novel, natural, and sustainable alternatives to conventional antibiotics and antifungal agents [ 1 ]. Essential oils (EOs), derived from aromatic plants, have garnered significant attention due to their broad-spectrum antimicrobial properties, low toxicity, and eco-friendly nature [ 2 ]. Among these, *Citrus limon* (L.) Osbeck EO (CLEO) has demonstrated promising antimicrobial activity, attributed to its rich composition of bioactive compounds, such as limonene, β-pinene, and γ-terpinene [ 3 ]. However, the practical application of CLEO is often limited due to its low water solubility, volatility, and susceptibility to degradation [ 4 ]. To overcome these challenges, nanotechnology-based delivery systems, particularly nanoemulsions (NEs), have emerged as a viable strategy to enhance the stability, bioavailability, and efficacy of EOs [ 5 ,  6 ].

NEs are colloidal dispersions consisting of oil, water, and surfactants, with droplet sizes typically ranging from 20 to 200 nm. Their small droplet size, high surface area, and thermodynamic stability make them ideal carriers for hydrophobic bioactive compounds, such as those found in CLEO. By encapsulation of CLEO in an NE, its antimicrobial properties can be potentiated, enabling targeted delivery and controlled release at the site of infection. Furthermore, NEs can improve the penetration of bioactive compounds through microbial cell membranes, enhancing their antimicrobial efficacy [ 7 ,  8 ].

Evaluation of the antibacterial and antifungal activities of CLEO NEs is of particular interest due to the growing demand for natural antimicrobial agents in various industries, including healthcare, food preservation, and agriculture. Previous studies have highlighted the antimicrobial potential of CLEO against a range of pathogens, including *Escherichia coli*, *Staphylococcus aureus*, *Candida albicans*, and *Aspergillus niger* [ 9 ]. However, the antimicrobial efficacy of CLEO NEs, particularly in comparison to its free oil form, remains underexplored. This study aimed to bridge this gap by systematically evaluating the antibacterial and antifungal activities of CLEO NEs against a panel of clinically relevant microorganisms.

While NEs offer promising advantages for enhancing the bioavailability and stability of bioactive compounds, potential toxicity and safety concerns must be carefully considered, particularly in healthcare and food applications. Recent studies have emphasized the need for rigorous toxicological evaluations to ensure biocompatibility and regulatory compliance, as physicochemical properties (e.g., particle size and surfactants) may influence cellular interactions and long-term safety [ 10 ].

Key components of CLEO, such as limonene, exert antimicrobial effects via membrane disruption and enzyme inhibition; however, their volatility and poor aqueous solubility limit their efficacy. These challenges are resolved by nanoemulsification through enhanced stability and bioavailability [ 11 ,  12 ].

In this context, the present study focused on the formulation and characterization of CLEO NEs, followed by an in-depth assessment of their antimicrobial activity. Findings of this *in vitro* study suggest that CLEO NEs may hold potential as a natural alternative to synthetic antimicrobial agents, though further validation is required to confirm their efficacy. By leveraging the synergistic benefits of nanotechnology and natural products, this study contributed to the ongoing efforts to combat antimicrobial resistance and promote sustainable solutions in antimicrobial therapy [ 13 ].

Enhanced antimicrobial efficacy of CLEO nanoemulsions positions them as promising candidates for clinical applications, such as adjunctive therapy for antifungal-resistant infections or topical management of dermatomycoses, where conventional treatments often face limitations [ 14 ].

## Materials and Methods

### Materials

The required EOs were purchased from Zardband Pharmaceuticals Co (Iran). Tween 20, and absolute ethanol were bought from Merck Company (Germany). Moreover, RPMI, penicillin-streptomycin, and DMSO (dimethyl sulfoxide) were provided by Shellmax Co. (China). Besides, FBS (Fetal bovine serum) was bought from Gibco Co. (USA). The deionized water was provided by the Central Laboratory of Mazandaran University of Medical Sciences, Sari, Iran. For antimicrobial evaluation, the Mueller Hinton Agar (MHA) culture medium was procured from Qlab (USA), and the Mueller Hinton Broth culture medium was obtained from Scharlau Company, Spain. Antibiotic discs for comparative analysis were sourced from Padtan Teb, Iran. All chemicals and reagents were of analytical grade and used without further purification. The microbial strains used for antibacterial and antifungal assays were obtained from standard culture collections and maintained according to recommended protocols.

### Microorganisms

The following microorganisms were used in the study: *S. aureus* (ATCC: 25923), *E. coli* (ATCC: 25922), *Staphylococcusepidermidis* (ATCC: 12228), *Enterococcusfaecalis* (ATCC: 29212), *A. fumigatus* (ATTC: 204304), *C. albicans* (ATTC: 90028) which were obtained from Invasive Fungi Research Center.  The bacterial strains and fungal strains were selected based on their clinical relevance, prevalence in healthcare-associated infections, and documented resistance to conventional antimicrobial agents. *E. faecalis* and *S. aureus* represent Gram-positive pathogens notorious for multidrug resistance (e.g., methicillin-resistant *Staphylococcus aureus* (MRSA) and vancomycin-resistant *enterococci* (VRE), while *E. coli* serves as a model Gram-negative bacterium with rising β-lactam resistance. *S.epidermidis* was included due to its role in biofilm-mediated device-related infections. The fungal strains, *C. albicans*, and *A. fumigatus* are leading causes of invasive mycoses and exhibit increasing azole resistance, underscoring the need for novel antifungal strategies [ 15 ,  16 ].

### Methods

### Preparation of nanoemulsion by spontaneous emulsification

For the synthesis of NEs, the low-energy spontaneous emulsification method was employed. The EO, surfactant (Tween 20), and co-surfactant (isopropyl alcohol) were initially mixed in different ratios using a magnetic stirrer and a specialized vial. The stirring speed was set at 1800 RPM, and the process was carried out for 30 min. Subsequently, distilled water (final volume 5 ml) was added gradually at room temperature to form the emulsions, and stirring was continued for an additional 10 min. The vials were then examined for stability and turbidity, and the transparent samples were selected for further analysis [ 17 ].

### Characterization of nanoemulsion by dynamic light scattering (DLS)

The droplet size, polydispersity index (PDI), and zeta potential of the CLEO were determined using DLS with a Zetasizer Nano ZS (Malvern Instruments, UK). The NE was diluted 1:10 with deionized water to avoid multiple scattering effects. Measurements were performed at 25 °C with a scattering angle of 173 °. The droplet size was reported as the Z-average diameter, while the PDI was used to assess the uniformity of droplet size distribution. The zeta potential was measured to evaluate the surface charge and stability of the NE. Stability of the NE was investigated at room temperature for one month and at times of 3, 7, 14, and 30 days, it was examined in terms of size, zeta potential, and polydispersity. All measurements were performed in triplicate, and the results were expressed as mean ± standard deviation [ 18 ].

### Chemical composition analysis by gas chromatography-mass spectrometry (GC-MS)

Chemical composition of CLEO and its NE was analyzed using gas chromatography-mass spectrometry (GC-MS). The analysis was performed on an Agilent Technologies GC-MS system equipped with an Rxi-5Sil MS capillary column (30 m × 0.25 mm × 0.25 µm). The oven temperature was programmed from 40 °C (held for 5 min) to 200 °C at a rate of 5 °C/min, and then 280 °C at a rate of 10 °C/min. Helium was used as the carrier gas at a flow rate of 1.0 mL/min. The injector and detector temperatures were set at 250 °C and 280 °C, respectively. The mass spectrometer was operated in electron ionization mode at 70 eV, with a scan range of 40–500 m/z. Identification of compounds was based on comparisons with the National Institute of Standards and Technology library and retention indices. The relative percentage of each compound was calculated from the peak area of the total ion chromatogram.

### Study of antimicrobial effects

The antimicrobial effects in the agar diffusion method were investigated using an MHA medium. In the method for determining the minimum inhibitory concentration (MIC), Mueller Hinton Broth liquid culture medium (Scharlau Company, Spain) was used, and Qulab MHA medium was employed to determine the minimum bactericidal concentration (MBC) for bacteria.

### Agar Diffusion Test

The agar diffusion test was conducted to evaluate the antimicrobial activity of lemon EO and its NE. Sterile paper discs (blank discs from Padten Teb, Iran) were impregnated with 10 µL of specific concentrations of the lemon EO extract and its NE. Sterile forceps were used to place each disc onto the surface of a solid agar medium inoculated with the test bacteria. The plates were then incubated at 37 °C for 48 h to allow the bacteria to reach full growth. For each test group, a total of 5 discs were used, along with 2 positive and 2 negative control discs. In this study, discs impregnated with streptomycin and gentamicin were used as positive controls, while blank discs (filter paper without any antimicrobial substance) served as negative controls for the tested microorganisms. After the incubation period, the diameter of any inhibition zones present on the plates was measured using a caliper and recorded. To ensure reliability, the experiment was repeated three times for each bacterial strain at different concentrations of the EO and antibiotics [ 19 ].

### Microdilution method for MIC and MBC determination

Antimicrobial properties of CLEO were evaluated using the microdilution method in a 96-well microplate, following CLSI guidelines. Antimicrobial Disk Susceptibility Tests were conducted according to the CLSI M100 guideline. The test bacteria were cultured in Mueller-Hinton broth for 18 h at 37 °C prior to the assay.

In the 96-well microplate, 72 µL of sterile Mueller-Hinton broth was added to six rows, and 100 µL was added to the remaining rows. To prepare serial dilutions, 128 µL of the CLEO or NE was added to the first six rows. A sterile pipette was used to transfer 100 µL from the first well to the second, and this process was repeated up to the eighth well. Three control wells were included: (1) a bacterial growth control (100 µL broth + 100 µL bacterial stock), (2) an antimicrobial control (100 µL broth + 100 µL NEor CLEO) to confirm sterility, and (3) a broth-only control.

After the preparation of the dilutions, 100 µL of the bacterial suspension was added to each well, except for the sterility control wells. The microplates were shaken at 250 rpm for 30 sec and incubated at 37 °C for 24 h. The MIC was determined as the lowest concentration of the NE or extract that inhibited visible bacterial growth. To determine the MBC, samples from wells showing no growth in the MIC range were streaked onto Mueller-Hinton agar plates and incubated at 37 °C for 24 h. The MBC was defined as the lowest concentration that killed 99.9% of the bacteria. All experiments were performed in duplicate to ensure accuracy [ 20 ].

### Microdilution broth method for antifungal activity assessment

The *in vitro* antifungal susceptibility testing was performed by broth microdilution according to the Clinical and Laboratory Standards Institute M27-A3/S4 and M38-A3 guidelines for *C. albicans* and *Aspergillus fumigatus*, respectively. Final concentration of antifungal drugs in the wells ranged from 0.016 to 16 µg/ml for voriconazole (Pfizer, Sandwich, UK), and 0.064-64 μg/mL for fluconazole (Pfizer, Groton, CT, USA). Lemon EO (Zardband Pharmaceuticals Co.) and its NE were prepared in RPMI, with concentrations ranging from 128 to 0.25 µg/mL. Stock solutions of agents were diluted in DMSO. Inoculum suspensions were prepared using saline containing 0.05% tween 20 and then adjusted spectrophotometrically at a wavelength of 530 nm to optical densities within the range of 75-77% for *C. albicans* and 80-82% for *A. fumigatus* and then diluted 1:100/1:50 in RPMI 1640 medium to obtain final inoculum between 0.5-2.5×10^3^ CFU/mL /ml for *Candida* and 0.4 × 10^4^ to 5 × 10^4^ CFU/ml for *Aspergillus* isolates. The 96-well plates were incubated at 35 °C in the dark and results were read visually after 24 and 48 h for *C. albicans* and *A. fumigatus*, respectively. The MIC was determined as the lowest concentration that completely inhibited the growth of fungi. The minimum fungicidal concentration (MFC) was defined as the lowest concentration that resulted in no fungal growth on the Sabouraud Dextrose Agar plates. *Candida krusei* (ATCC 6258), *Candida parapsilosis* (ATCC 22019), and *Aspergillus flavus* (ATCC 2004304) served as quality control strains. All experiments were performed in duplicate to ensure reproducibility [ 21 - 23 ].

### Statistical analysis

In this study, statistical analysis was performed using SPSS software (version 21). The independent t-test was employed to compare the means between groups, with a significance level set at *p* < 0.05 to determine statistical significance. The results were interpreted based on the p values obtained from the t-test analysis.

### Ethics approval

The current study was approved by the Ethics Committee of the Mazandaran University of Medical Sciences, Sari, Iran (IR.MAZUMS.REC.1398.487).

## Results

### GC-mass analysis of Citrus limon (L.) Osbeck essential oil

Chemical composition of CLEO was analyzed using GC-MS. The GC-MS chromatogram revealed the presence of several bioactive compounds, with limonene identified as the predominant constituent, accounting for approximately 48.93% of the total oil composition. Other significant compounds included α-Pinene (4.51%), γ-terpinene (8.73%), 3-Carene (17.65%), β–Myrcene (1.54%), and β -Citral (1.81%). Minor constituents, such as linalool, were also detected, contributing to the overall bioactivity of the oil (Table 1). The identified compounds were consistent with the known phytochemical profile of CLEO and were likely responsible for its antimicrobial properties. These findings provide a comprehensive understanding of the chemical basis for the observed biological activities of CLEO and its NE.

**Table 1:** List of lemon essential oil compounds.

Num	Compounds	Retention Time (min)	KI	Percentage	Type of Compounds
1	α-Pinene	8.765	933	4.51	Monoterpene
2	3-Carene	10.665	1004	17.65	Monoterpene
3	β-Myrcene	11.079	1176	1.54	Monoterpene
4	Limonene	13.107	1234	48.93	Monoterpene
5	γ-Terpinene	13.853	1265	8.73	Monoterpene
6	β-Citral	19.140	1240	1.81	Monoterpenoid

### Characterisation of nanoemulsion

**Figure 1:**
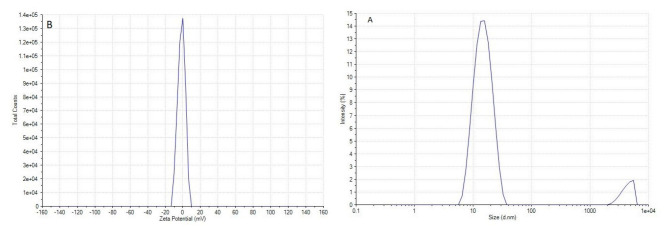
A) Particle size of *Citrus limon* (L.) Osbeck essential oil nanoemulsions. B) Zeta potentials of *Citrus limon* (L.) Osbeck essential oil nanoemulsions

Nanoemulsion of CLEO was successfully prepared using the spontaneous emulsification method. DLS analysis revealed that the resulting particles had an average size of 15 nm, indicating the formation of a finely dispersed NE. The PDI was 0.274, confirming a narrow and uniform size distribution. The zeta potential of the NE was measured at 1.64 mV, suggesting moderate stability due to electrostatic repulsion between droplets (Figure 1). Furthermore, the NE demonstrated excellent stability when stored at ambient temperature for one month, with no significant changes in particle size, PDI, or zeta potential. These results highlight the potential of the CLEO NE as a stable and effective delivery system for antimicrobial applications.

### Antimicrobial activity of lemon essential oil

The antimicrobial effects of lemon EO were evaluated using the agar diffusion method. Concentrations of 1, 2, 4, 8, 16, 32, and 64 µg/mL of the EO were tested and compared with gentamicin and streptomycin. The results revealed that *E. faecalis* was the most sensitive bacterium among those tested, showing a 10 mm zone of inhibition at a concentration of 2 µg/mL, while no inhibition zones were observed for the other bacteria at this concentration. The highest antibacterial activity was observed at 64 µg/mL, where the largest inhibition zones were recorded. In contrast, *E. coli* was the most resistant bacterium in this study, displaying the smallest inhibition zones across all tested concentrations. These findings highlight the varying susceptibilities of the tested bacteria to lemon EO, with *E. faecalis* being the most sensitive and *E. coli* the most resistant (Table 2).

**Table 2:** Average diameter of the growth inhibition zone (mm) at different dilutions of essential oil and antibiotics (the data are presented as mean ±SD (n = 3)

Type of Bacteria	Staphylococcus aureus	Staphylococcus epidermidis	Escherichia coli	Enterococcus faecalis
Concentration (µg/ml)				
64	28.6 ± 2.5	23.1 ± 1.6	20.3 ± 2.1	33.2 ± 3.1
32	22.0 ± 1.7	19.6 ± 1.1	17.1 ± 1.3	27.1 ± 1.7
16	16.4 ± 1.1	15.2 ± 1.0	14.5 ± 0.9	21.4 ± 1.2
8	11.9 ± 0.6	12.0 ± 0.8	10.0 ± 0.7	15.6 ± 0.8
4	9.2 ± 0.5	8.5 ± 0.9	-	13.2 ± 0.6
2	-	-	-	10.2 ± 0.4
1	-	-	-	-
Gentamicin10	-	17.9 ± 1.1	23 ± 1.9	24.3 ± 1.4
Streptomycin10	15.0 ± 1.0	7.3 ± 1.3	12.0 ± 0.7	14.2 ± 0.8

Inhibitory effects of CLEO were further evaluated using the microdilution method to determine the MIC and MBC. According to the results, *E. coli* was identified as the most resistant bacterium, with an MIC of 12.5 µg/mL. In contrast, *S. aureus* and *S. epidermidis* exhibited a MIC value of 6.25 µg/mL, while *E. faecalis* showed the lowest MIC value of 3.12 µg/mL, confirming its high sensitivity to lemon EO.

Regarding the MBC, *E. coli* required the highest concentration of 25 µg/mL to achieve bactericidal effects. For *S. aureus* and *S. epidermidis*, the MBC was 12.5 µg/mL, and for *E. faecalis*, it was 6.25 µg/mL. These results further emphasize the varying susceptibilities of the tested bacteria, with *E. faecalis* being the most sensitive and *E. coli* the most resistant to the antimicrobial effects of lemon EO.

### Antimicrobial effects of lemon nanoemulsion

Antimicrobial effects of the prepared lemon NE were evaluated using the agar diffusion method at various concentrations and compared with the positive control groups (gentamicin and streptomycin). The results demonstrated that the NE exhibited superior antimicrobial activity, compared to the raw lemon EO at all tested concentrations (*p* < 0.05). At the highest concentration, which was 64 µg/mL, the NE showed the most significant antibacterial effects, even outperforming the positive controls (gentamicin and streptomycin), (Table 3).

**Table 3:** Average diameter of the growth inhibition zone (mm) at different dilutions of *Citrus limon* (L.) Osbeck essential oil nanoemulsion and antibiotics (The data are presented as mean ±SD (n = 3)

Type of Bacteria	*Staphylococcus aureus*	*Staphylococcus epidermidis*	*Escherichia coli*	*Enterococcus faecalis*
Concentration (µg/ml)				
64	30.7 ± 2.8	27.3 ± 1.3	24.3 ± 1.8	35.6 ± 2.7
32	25.6 ± 1.6	23.8 ± 1.2	20.0 ± 1.1	31.9 ± 2.2
16	17.1 ± 1.2	16.1 ± 0.8	15.4 ± 0.8	26.4 ± 1.4
8	14.8 ± 0.9	13.5 ± 1.0	11.0 ± 0.9	18.3 ± 1.0
4	11.5 ± 0.6	10.1 ± 0.6	8.4 ± 0.7	16.3 ± 1.5
2	-	-	-	12.2 ± 0.7
1	-	-	-	-
Gentamicin10	-	17.9 ± 1.1	23 ± 1.9	24.3 ± 1.4
Streptomycin10	15.0 ± 1.0	7.3 ± 1.3	12.0 ± 0.7	14.2 ± 0.8

All tested bacteria exhibited greater sensitivity to the NE, compared to the raw EO (*p* < 0.05). This enhanced efficacy highlights the potential of the NE formulation in improving the antimicrobial properties of lemon EO, making it a promising candidate for further applications in antimicrobial therapies.

The antimicrobial activity of CLEO NE was evaluated using MIC and MBC assays. The NE was tested at concentrations ranging from 50 to 0.1 μg/mL against various bacterial strains.

Much data has been collected from this test, the results of which are summarized as follows. Among the bacteria examined, *E. coli* and *S. epidermidis* exhibited higher resistance to the lemon NE, compared to other strains. The MIC values for *E. coli* and *S. epidermidis* were determined at 0.78 μg/mL, while their MBC value was 1.56 μg/mL.These results indicate that the lemon NE demonstrates significant antimicrobial activity against the tested bacterial strains, with *E. coli* and *S. epidermidis* requiring slightly higher concentrations for inhibition and bactericidal effects. Effectiveness of NE against these common pathogens suggests its potential application in various antimicrobial treatments. The NE demonstrated enhanced antibacterial activity, compared to gentamicin and streptomycin at equivalent mass concentrations (µg/mL), though molarity-adjusted comparisons are warranted to further validate these findings.

### Results of antifungal activity of lemon essential oil

Antifungal effects of lemon EO and NE were evaluated against *A. fumigatus* and *C. albicans*. To assess the antifungal activity, MIC tests were conducted using concentrations ranging from 800 to 1.56 μg/mL.The results demonstrated significant antifungal activity of lemon EO against both fungal species. For Aspergillus fumigatus, the MIC was determined at 6.25 μg/mL. For *C. albicans*, the MIC was found to be 3.12 μg/mL (Table 3).

These findings indicate that lemon EO exhibits potent antifungal properties, with a notably stronger inhibitory effect against *C. albicans*, compared to *A. fumigatus*. The lower MIC value for *C. albicans* suggests that it is more susceptible to the antifungal components present in lemon EO [ 24 ]. Additionally, the MFC of lemon EO was determined for these two fungal species, which were obtained at 12.5 μg/mL for *A. fumigatus* and 6.25 μg/mL for *C. albicans*.

### Results of antifungal activity of CLEO nanoemulsion

The method described previously for measuring MIC and MFC was applied to lemon NE, with concentrations evaluated down to 0.1 μg/mL. The MIC of the NE were found to be 0.39 μg/mL for *A. fumigatus* and 0.1 μg/mL for *C. albicans*. The MFC values were 0.78 μg/mL for *A. fumigatus* and 0.195 μg/mL for *C. albicans*. Fluconazole and voriconazole showed lower MIC and MFC values against *C. albicans* and *A. fumigatus*, respectively, compared to CLEO, while these antifungal agents demonstrated higher MIC and MFC values than NE (Table 4). Therefore, these results emphasize the superior efficacy of NE, compared to fluconazole and voriconazole (*p* < 0.05).

**Table 4:** Antifungal potencies of Citrus limon (L.) Osbeck essential oil (CLEO) and nanoemulsion (NE) against the selected fungi; minimum inhibitory concentration (MIC) and minimum fungicidal concentration (MFC) values are depicted as µg/mL.

Fungal species	CLEO Concentration(µg/ml)		NE Concentration(µg/ml)		Fluconazole Concentration (µg/ml)		Voriconazole Concentration (µg/ml)	
	MIC	MFC	MIC	MFC	MIC	MFC	MIC	MFC
*Candida albicans*	3.12	6.25	0.1	0.195	1	4	-	-
*Aspergillus fumigatus*	6.25	12.5	0.39	0.78	-	-	0.5	1

## Discussion

Comparative evaluation of the antimicrobial and antifungal activities of lemon EO and its NE revealed significant differences in efficacy, with the NE demonstrating superior performance. The NE, with an average particle size of approximately 15 nm, exhibited enhanced antimicrobial and antifungal effects, compared to the raw EO. This improvement can be attributed to several factors, including the increased surface area, improved stability, and enhanced bioavailability of the NE, which facilitate better interaction with microbial and fungal cell membranes [ 14 ].

The low zeta potential (−1.64 mV) suggests limited electrostatic stabilization, which is atypical for stable emulsions. However, the observed long-term stability is attributed to the steric hindrance provided by Tween 20, which prevents droplet aggregation despite weak electrostatic repulsion. This is aligned with mechanisms reported in systems utilizing non-ionic surfactants [ 25 ].

The study demonstrates that CLEO exhibits concentration-dependent antimicrobial activity, with notable efficacy against *E. faecalis* and limited effects on *E. coli*. The NE formulation significantly enhanced antimicrobial potency, surpassing both raw CLEO and conventional antibiotics (gentamicin/streptomycin) at higher concentrations. This improvement is likely attributed to the increased surface area and stability of NE, facilitating better bacterial membrane penetration. The variability in bacterial susceptibility—*E. faecalis* being highly sensitive versus *E. coli* being resistant—may reflect differences in cell wall structures or efflux mechanisms. Remarkably, the NE achieved reduced MIC/MBC values (e.g., 0.78 µg/mL for *E. coli*), underscoring its potential for optimized antimicrobial delivery. These findings advocate for nanoemulsified CLEO as a promising alternative in combating resistant pathogens, warranting further exploration in clinical and industrial contexts.

Antifungal activity of lemon EO and its NE was evaluated against *A. fumigatus* and *C. albicans*, revealing significant differences in their efficacy. The raw EO demonstrated antifungal activity with MIC values of 6.25 µg/mL for *A. fumigatus* and 3.12 µg/mL for *C. albicans*. The MFC values were 12.5 µg/mL and 6.25 µg/mL for *A. fumigatus* and *C. albicans*, respectively. However, the NE formulation showed even greater antifungal efficacy, with both MIC and MFC values significantly lower than those of the raw EO. This enhancement in antifungal activity can be attributed to the unique properties of the NE, including its small particle size (approximately 15 nm), improved stability, and increased bioavailability [ 26 ].

The observed antifungal activity of CLEO aligns with the results of previous studies performed on the antimicrobial properties of citrus-derived EOs. The effectiveness against these common fungal pathogens highlights the potential of lemon EO as a natural antifungal agent [ 27 ].

These results contribute to the growing body of evidence supporting the use of natural compounds, particularly EOs, in combating fungal infections. This study demonstrates that lemon EO could be a promising alternative or complementary treatment for infections caused by *A. fumigatus* and *C. albicans*, especially in the context of increased resistance to conventional antifungal drugs.

The superior antifungal performance of the NE is likely due to its ability to penetrate fungal cell walls and membranes more effectively than the raw EO. The small particle size of the NE allows for better interaction with fungal cells, leading to increased membrane disruption and cell lysis. Additionally, the encapsulation of EO components by NE ensures a sustained release, prolonging the contact time with fungal cells and enhancing its antifungal effects. This mechanism aligns with previous studies that have reported improved antifungal activity of NEs due to their ability to destabilize fungal membranes and increase permeability [ 28 ,  29 ]. Finally, the structure of the formulation also protects bioactive oil components (e.g., Limonene) from oxidative/enzymatic degradation, prolonging their antifungal activity [ 30 ].

The results of this study clearly demonstrate that the NE formulation significantly enhances the antifungal activity of lemon EO. For both *A. fumigatus* and *C. albicans*, the MIC and MFC values for the NE were lower than those for the raw EO. This improvement is consistent with the findings of other researchers who have reported that NEs of EOs exhibit higher antifungal efficacy due to their improved solubility, stability, and ability to interact with fungal cells. For example, Quatrin *et al*. (2017) reported that NEs of eucalyptus oil exhibited enhanced antifungal activity against *C. albicans* due to their ability to disrupt fungal cell membranes [ 31 ]. Similarly, Li *et al*. (2016) found that NEs of tea tree oil showed improved antifungal effects against fungal pneumonia after pulmonary inhalation [ 32 ]. Our results further validate these observations, highlighting the potential of NEs as effective antifungal agents.

The enhanced antifungal activity of the lemon EO NE makes it a promising candidate for various applications, including the treatment of fungal infections, food preservation, and agricultural antifungal agents. Its ability to achieve lower MIC and MFC values, compared to the raw EO suggests its potential as an alternative or complementary treatment for fungal infections caused by *A. fumigatus* and *C. albicans*. Findings of the present research align with those of a study performed by Chen *et al*., which documented the antimicrobial efficacy of lemon oil in food systems. Building on this foundation, our NE not only preserves these inherent properties but enhances antibacterial potency through improved bioavailability and stability, addressing key limitations of raw oil [ 6 ].

Given their potency against resistant pathogens, these NEs could be explored for translational use in wound care, oral thrush management, or as combinatorial agents to reduce reliance on traditional antifungals, pending further *in vivo* and clinical validation [ 33 ]

While this study focused on evaluating antimicrobial efficacy, future works need to prioritize cytotoxicity and hemocompatibility assessments to ensure the safety of CLEO NEs in clinical or therapeutic contexts. Previous studies have highlighted the importance of such evaluations for NE formulations, as surfactant choice and particle size may influence biocompatibility [ 34 ,  35 ].

While this study demonstrated promising antifungal efficacy of the NE, certain limitations should be noted. First, all experiments were conducted *in vitro*, and further *in vivo* studies are required to evaluate the behavior of formulation in biological systems, including bioavailability and immune interactions. Second, the tested fungal strains were non-resistant laboratory isolates; future work should assess efficacy against clinically resistant strains to better reflect real-world challenges. Finally, cytotoxicity and long-term safety profiles of the NE remain uncharacterized, which is critical for translational applications. Addressing these gaps will be a focus of subsequent research.

## Conclusion

In conclusion, while the NE formulation of lemon EO suggested enhanced antifungal potential against A. fumigatus and C. albicans (demonstrated by reduced MIC/MFC values *in vitro*), the primary contribution of this study lies in its antibacterial efficacy, supported by comprehensive physicochemical characterization. The observed improvements in antifungal activity, potentially linked to the small particle size and stability of NE, remain preliminary and require validation in biological systems. Importantly, these findings align with broader evidence on NEs as promising delivery systems for EOs. Future research must prioritize *in vivo* efficacy testing, safety assessments, and evaluation against clinically resistant strains to confirm translational relevance. This study also underscores the need for cautious interpretation of *in vitro* antifungal data while highlighting actionable pathways for advancing NE-based therapies.

## References

[1] Helmy YA, Taha-Abdelaziz K, Hawwas HAE, Ghosh S, AlKafaas SS, Moawad MMM, et al ( 2023). Antimicrobial resistance and recent alternatives to antibiotics for the control of bacterial pathogens with an emphasis on foodborne pathogens. Antibiotics (Basel)..

[2] Medeleanu ML, Farcas AC, Coman C, Leopold L, Diaconeasa Z, Socaci SA ( 2023). Citrus essential oils - Based nano-emulsions: functional properties and potential applications. Food Chem X.

[3] Ben Hsouna A, Ben Halima N, Smaoui S, Hamdi N ( 2017). Citrus lemon essential oil: chemical composition, antioxidant and antimicrobial activities with its preservative effect against Listeria monocytogenes inoculated in minced beef meat. Lipids Health Dis.

[4] Tanasă F, Nechifor M, Teacă C-A ( 2024). Essential oils as alternative green broad-spectrum biocides. Plants.

[5] Valipour S, Ebrahimzadeh MA, Mobini GR, Akhtari J ( 2017). The use of nanoparticles in the formulation of essential oils. J Shahrekord Univ Med Sci.

[6] Chen C, Meng F-B, Lv H-J, Gou Z-Z, Qiu J, Li Y-C ( 2024). Study on the bacteriostasis of lemon essential oil and the application of lemon essential oil nanoemulsion on fresh-cut kiwifruit. Front Sustain Food Syst.

[7] Elzayat A, Adam-Cervera I, Alvarez-Bermudez O, Munoz-Espi R ( 2021). Nanoemulsions for synthesis of biomedical nanocarriers. Colloids Surf B Biointerfaces.

[8] Barradas TN, de Holanda e Silva KG ( 2021). Nanoemulsions of essential oils to improve solubility, stability and permeability: a review. Environ Chem Lett.

[9] Viuda‐Martos M, Ruiz‐Navajas Y, Fernandez‐Lopez J, Perez‐Álvarez J ( 2008). Antibacterial activity of lemon (Citrus lemon L.), mandarin (Citrus reticulata L.), grapefruit (Citrus paradisi L.) and orange (Citrus sinensis L.) essential oils. J Food Safety.

[10] McClements DJ, Das AK, Dhar P, Nanda PK, Chatterjee N ( 2021). Nanoemulsion-based technologies for delivering natural plant-based antimicrobials in foods. Front Sustain Food Syst.

[11] Burt S ( 2004). Essential oils: their antibacterial properties and potential applications in foods—a review. Int J Food Microbiol.

[12] Tan C, McClements DJ ( 2021). Application of advanced emulsion technology in the food industry: A review and critical evaluation. Foods.

[13] Enrico C ( 2019). Nanotechnology-based drug delivery of natural compounds and phytochemicals for the treatment of cancer and other diseases. Studies Natural Products Chem.

[14] Garcia CR, Malik MH, Biswas S, Tam VH, Rumbaugh KP, Li W, et al ( 2022). Nanoemulsion delivery systems for enhanced efficacy of antimicrobials and essential oils. Biomaterials Sci.

[15] Pappas PG, Lionakis MS, Arendrup MC, Ostrosky-Zeichner L, Kullberg BJ ( 2018). Invasive candidiasis. Nat Rev Dis Primers.

[16] Fisher MC, Alastruey-Izquierdo A, Berman J, Bicanic T, Bignell EM, Bowyer P, et al ( 2022). Tackling the emerging threat of antifungal resistance to human health. Nat Rev Microbiol.

[17] Rasti F, Ahmadi E, Safari M, Abdollahi A, Satvati S, Ranjbar R, et al ( 2023). Anticancer, antioxidant, and antibacterial effects of nanoemulsion of Origanum majorana essential oil. Iranian J Microbiol.

[18] Amin N, Das B ( 2019). A review on formulation and characterization of nanoemulsion. Int J Curr Pharm Res.

[19] Krishnamoorthy R, Athinarayanan J, Periasamy VS, Adisa AR, Al-Shuniaber MA, Gassem MA, et al ( 2018). Antimicrobial activity of nanoemulsion on drug-resistant bacterial pathogens. Microb Pathog.

[20] Ranjbar R, Zarenezhad E, Abdollahi A, Nasrizadeh M, Firooziyan S, Namdar N, et al ( 2023). Nanoemulsion and nanogel containing Cuminum cyminum L essential oil: antioxidant, anticancer, antibacterial, and antilarval properties. J Trop Med.

[21] Haghani I, Hashemi SM, Abastabar M, Yahyazadeh Z, Ebrahimi-Barough R, Hoseinnejad A, et al ( 2025). In vitro and silico activity of piperlongumine against azole-susceptible/resistant Aspergillus fumigatus and terbinafine-susceptible/resistant Trichophyton species. Diagn Microbiol Infect Dis.

[22] Kermani F, Sadeghian M, Shokohi T, Hashemi S, Moslemi D, Davodian S, et al ( 2021). Molecular identification and antifungal susceptibility testing of Candida species isolated from oral lesions in patients with head and neck cancer undergoing radiotherapy. Curr Med Mycol.

[23] Haghani I, Akhtari J, Yahyazadeh Z, Espahbodi A, Kermani F, Javidnia J, et al ( 2023). Potential inhibitory effect of miltefosine against terbinafine-resistant Trichophyton indotineae. Pathogens (Basel, Switzerland)..

[24] Jing L, Lei Z, Li L, Xie R, Xi W, Guan Y, et al ( 2014). Antifungal activity of citrus essential oils. J Agric Food Chem.

[25] Tran E, Richmond GL ( 2021). Interfacial steric and molecular bonding effects contributing to the stability of neutrally charged nanoemulsions. Langmuir.

[26] Moazeni M, Davari A, Shabanzadeh S, Akhtari J, Saeedi M, Mortyeza-Semnani K, et al ( 2021). In vitro antifungal activity of Thymus vulgaris essential oil nanoemulsion. J Herbal Med.

[27] Van Hung P, Chi PT, Phi NT ( 2013). Comparison of antifungal activities of Vietnamese citrus essential oils. Nat Prod Res.

[28] Perumal AB, Li X, Su Z, He Y ( 2021). Preparation and characterization of a novel green tea essential oil nanoemulsion and its antifungal mechanism of action against Magnaporthae oryzae. Ultrason Sonochem.

[29] Pongsumpun P, Iwamoto S, Siripatrawan U ( 2020). Response surface methodology for optimization of cinnamon essential oil nanoemulsion with improved stability and antifungal activity. Ultrason Sonochem.

[30] Lohith Kumar D, Sarkar P ( 2018). Encapsulation of bioactive compounds using nanoemulsions. Environ Chem Lett.

[31] Quatrin PM, Verdi CM, de Souza ME, de Godoi SN, Klein B, Gundel A, et al ( 2017). Antimicrobial and antibiofilm activities of nanoemulsions containing Eucalyptus globulus oil against Pseudomonas aeruginosa and Candida spp. Microb Pathogen.

[32] Li M, Zhu L, Liu B, Du L, Jia X, Han L, et al ( 2016). Tea tree oil nanoemulsions for inhalation therapies of bacterial and fungal pneumonia. Colloids Surf B Biointerfaces.

[33] Nazzaro F, Fratianni F, Coppola R, Feo V ( 2017). Essential oils and antifungal activity. Pharmaceuticals (Basel)..

[34] McClements DJ, Jafari SM ( 2018). General aspects of nanoemulsions and their formulation.

[35] Gupta A ( 2020). Nanoemulsions.

